# Drivers of Time-Activity Budget Variability during Breeding in a Pelagic Seabird

**DOI:** 10.1371/journal.pone.0116544

**Published:** 2014-12-31

**Authors:** Gavin M. Rishworth, Yann Tremblay, David B. Green, Maëlle Connan, Pierre A. Pistorius

**Affiliations:** 1 DST/NRF Centre of Excellence at the Percy FitzPatrick Institute, Department of Zoology, Nelson Mandela Metropolitan University, Summerstrand, South Africa; 2 Institut de Recherche pour le Développement (IRD), UMR EME-212 Exploited Marine Ecosystems, Centre de Recherche Halieutique Méditerranéenne et Tropicale, Sète cedex, France; 3 Instituto del mar del Peru (IMARPE), Esquina Gamarra y gal Valle s/n Chucuito Callao, Peru; Hokkaido University, Japan

## Abstract

During breeding, animal behaviour is particularly sensitive to environmental and food resource availability. Additionally, factors such as sex, body condition, and offspring developmental stage can influence behaviour. Amongst seabirds, behaviour is generally predictably affected by local foraging conditions and has therefore been suggested as a potentially useful proxy to indicate prey state. However, besides prey availability and distribution, a range of other variables also influence seabird behavior, and these need to be accounted for to increase the signal-to-noise ratio when assessing specific characteristics of the environment based on behavioural attributes. The aim of this study was to use continuous, fine-scale time-activity budget data from a pelagic seabird (Cape gannet, *Morus capensis*) to determine the influence of intrinsic (sex and body condition) and extrinsic (offspring and time) variables on parent behaviour during breeding. Foraging trip duration and chick provisioning rates were clearly sex-specific and associated with chick developmental stage. Females made fewer, longer foraging trips and spent less time at the nest during chick provisioning. These sex-specific differences became increasingly apparent with chick development. Additionally, parents in better body condition spent longer periods at their nests and those which returned later in the day had longer overall nest attendance bouts. Using recent technological advances, this study provides new insights into the foraging behaviour of breeding seabirds, particularly during the post-guarding phase. The biparental strategy of chick provisioning revealed in this study appears to be an example where the costs of egg development to the female are balanced by paternal-dominated chick provisioning particularly as the chick nears fledging.

## Introduction

Animals face a trade-off in time-allocation within their activity budgets which is dependent on their current state (e.g. breeding) as well as external pressures (e.g. predation risk) [1], [2]. While breeding, they are especially sensitive to environmental and resource availability [3]. This is apparent in seabirds, which are long-lived species, where the costs of reproduction are balanced against future survival [1], [4]. Poor conditions in terms of prey availability are often reflected through reduced breeding success (e.g. [5]). Amongst animals such as seabirds which exhibit biparental care [6], disproportionate reproductive investment from one partner might require compensation by the other to maximise offspring fitness (e.g. [7]). Consequently, differing sexes have evolved traits which limit competition for similar resources while provisioning their offspring [8], [9]. Strategies such as divergent foraging locations (e.g. [8]), physical dimorphism allowing for or forcing separate resource niches (e.g. [10]), or paternal dominance of offspring provisioning to compensate for female investment in egg or foetus development (e.g. [11]) appear to reduce inter-sex competition for prey resources. In terms of offspring survival, accommodating these sex-specific behaviours is important for seabird conservation to ensure that the resource requirements of both parents are met [12], [13].

Besides resource availability and gender roles, other factors also influence how seabirds allocate their time during breeding. In order to survive, adults in poor condition tend to spend more time foraging at the cost to offspring allocation [14], [15]. The pre-breeding season is therefore an important preparation period for reproduction [16]. Seabirds which have not built up sufficient reserves during the non-breeding period often exhibit reduced reproductive success [17]. These factors, together with heritable or learnt traits, affect the amount of inter-individual variability [18], [19], which can be as important as inter-sex differences in driving behavioural variability [20]. Data on an animal's time-activity budget in relation to these factors that potentially influence behaviour are important when interpreting their responses to external factors and the state of the environment [21], [22]. Therefore, the aim of this study was to investigate which variables influence parental time-activity budgets while breeding in a seabird. We selected the colonially-breeding Cape gannet, *Morus capensis*, as a model species for several ecological and practical reasons:

1. The Cape gannet is a biparental income breeder, making the trade-offs in resource allocation between the developing offspring and each parent a dynamic state [7]. During the breeding season (i.e. September to April) parents alternate nest attendance during both incubation and brooding (up to c.a. 40 days old [23]), spending more time simultaneously away from the nest as the chick ages [5], [24]. Additionally, there is some evidence for sex-specific foraging behaviour in this monomorphic seabird while brooding [5], [25] although the ecological significance of this amongst gannets is not clear [8].
2. Understanding the constraints placed on adults throughout offspring development is important [1], and being a long-lived species, poor resource availability will be reflected in terms of reproduction rather than adult survival in the Cape gannet [5], [26]. This study builds upon the few studies which have investigated time-activity budgets in volant seabirds during the post-guarding phase of chick development [24], [27].
3. The Cape gannet is a colonial-breeder [28] and central-place forager [29], with strong nest-site fidelity [30], which offers several advantages in terms of ease, quantity and quality of data collection (see ‘Materials and Methods’ and [24]).
4. Lastly, from a fisheries management perspective, seabird behavioural data have been advocated as a useful proxy for prey conditions [31], [32] as the time-activity budget of many seabirds is sensitive to fluctuating prey availability [33]–[36]. However, the correlation between seabird behaviour and prey availability can be masked by factors such as gender [9], [37], individual variability in phenotypes [38], conspecific and interspecific interactions [7], [39]–[41], chick age [42], time constraints [43], individual responses [18], [20], and meteorological conditions [44]. It is consequently important to understand how these factors relate to seabird behaviour. The Cape gannet has been suggested as a potential biomonitor of its local environment [45] as the effects of variability in its two primary, and commercially-important, prey items, sardine, *Sardinops sagax* and anchovy, *Engraulis encrasicolus* [46], [47], have been demonstrated [26], [48].

Using a new generation VHF-based monitoring system that recorded data on time-activity budgets of Cape gannet breeding pairs throughout the breeding season [24], we investigated the influence of chick age, sex, time of arrival or departure to or from the nest and body condition on a number of adult behavioural parameters. In particular we were interested in foraging trip duration, nest attendance and trip frequency as response variables.

## Materials and Methods

### Ethics statement

All fieldwork and data collection was undertaken under the ethics clearance reference A10-SCI-ZOO-008 issued by the Research Ethics Committee at the Nelson Mandela Metropolitan University. All fieldwork was conducted in accordance with the strict recommendations of South African National Parks (SANParks), as approved by the SANParks Animal Use and Care Committee (AUCC). The devices attached to our study birds did not negatively affect chick growth-rates or fledging success, nor adult foraging trip or nest attendance durations [24].

### Data collection

Pairs of breeding Cape gannets from 20 and 30 nests on Bird Island, Algoa Bay (33° 50′S, 26° 17′E), were fitted with VHF transmitters (NTQB-6-2, Lotek Wireless, United Kingdom) in the 2011/2012 (10–15 December 2011) and 2012/2013 (5–12 December 2012) breeding seasons, respectively to automatically record their presence at the island (see [24]). Transmitters were attached to PVC leg-rings and with the rings weighed ∼10 g, approximately 0.4% of the average body mass of Cape gannets measured during this study. A coded signal was transmitted at 150.38 MHz every 39–40 s from the transmitters and received by a Yagi antenna fitted to a 12 V solar-powered receiver (DataSika-C5; BioTrack, United Kingdom) when birds were at their nests. Received signals were recorded as a unique coded identity in addition to a date, time and signal strength. Data from the VHF receiver were downloaded on a monthly basis during both breeding seasons.

Body mass (to the nearest 25 g) and wing chord length (to the nearest 1 mm) were measured at deployment from each equipped bird. Body condition of adults was expressed as body mass over wing length [45]. A few breast feathers were plucked from each bird to allow for DNA sex-determination [49]. All parents fitted with VHF transmitters were attending a chick no younger than one week old. Each chick was carefully removed from its nest to measure culmen length (to the nearest 0.1 mm), wing chord length and body mass and returned within three minutes. The age of each chick at the time of its parent's VHF transmitter attachment was determined from morphometric measurements [50]: when wing chord length was less than 40 mm, age (days) = −ln((89.78−*b*/6.15×*b*)/0.086)+0.5, and when it was greater than 40 mm, age = 1.395−ln(ln(588.8/*w*)/0.0264)+0.5, where *b* is culmen length (mm) and *w* is wing chord length (mm). To aid identification, unique PVC leg-rings were fitted to chicks when their tarsus had a large enough diameter (older than 3 weeks). Nests were periodically checked during the breeding season (8–28 December, 27 January to 6 February and 22–26 March 2011/2012 and 5–13 December, 15–27 January and 27 February 2012/2013) and all chick mortalities were recorded.

### Data analysis

All data recorded using the VHF receiver were converted into trip durations using a purpose-built (YT, unpubl.) interface in MatLab (R2011a; MathWorks, United States of America) [24]. Data were imported at a 10-minute resolution into the MatLab interface, using 10-minute bins as a level of precision corresponding to approximately three minutes longer than the receiver's scan frequency. Nest attendance duration, strictly implying attendance duration at the colony while within the receiver's range, and trip frequency per day (hereafter referred to as provisioning rate, exclusive of food delivery rates) were also calculated. Data used for the purposes of this study were restricted to the period up to when the respective chicks either died or fledged or until the transmitter ceased functioning (see [24]). If the chick fledged (no mortality recorded) then the data were selected until the chick was 100 days old, the average fledging age [51].

Foraging trip and nest attendance duration as well as provisioning rate were all right-skewed and therefore log-transformed before each was incorporated into a linear mixed-effects model (LMM; “lmer” in the “lme4” package) fitted by restricted maximum likelihood (REML) using R (R 2.15.1; R Foundation for Statistical Computing, Vienna, Austria). The following predictor variables were used: chick age (as a continuous number), sex, adult body condition and time of behaviour initiation (foraging trip or nest attendance) as a factor grouped into one-hour bins as well as the interaction of sex with each of these variables. All permutations of the predictor variables for each adult behavioural parameter were modelled separately and Akaike Information Criterion (AIC) scores [52] calculated. The most parsimonious model had the lowest AIC score with other models having ΔAIC≤2 also being considered [53]. To account for the effect of repeated measures (pseudoreplication), nest-site (which accounted for potential correlation of partner behaviour during chick brooding) and individual were specified in the model as random intercepts. The significance of the effect of inter-seasonal variability was tested using log-likelihood ratio tests (LRT [54]), comparing the null model (no explanatory variables included) to the null plus random intercept model. A significance level of α = 0.05 was used and all results are presented as mean ±95% CI of the mean.

## Results

The VHF receiver on Bird Island recorded 4,563 foraging trips and 4,463 nest attendance bouts from VHF-equipped Cape gannets in the 2011/2012 (n = 1,108 and 1,068, respectively) and 2012/2013 (n = 3,455 and 3,395, respectively) breeding seasons. Chicks were measured from a younger age in 2012/2013 (18.6±2.6 versus 24.8±2.7 d; t_(2,48)_ = 3.14, p<0.05) as nest monitoring began approximately five days earlier than in 2011/2012.

Parental behaviour parameters (foraging trip duration, nest attendance duration and provisioning rate) were not significantly different between the two breeding seasons (LRTs: all p>0.10) and therefore data from both seasons were pooled. Foraging trip duration was best explained by chick age, adult sex and the interaction of adult sex with chick age (Model T1; Table 1). Nest attendance duration was best predicted by chick age, nest arrival time and adult body condition (Models N1 and N2; Table 1). Chick age and adult sex were the best predictors of adult provisioning rate (Models Tf1 and Tf2; Table 1). Residual errors for these models appeared normally distributed. Interaction predictors of adult sex with both time and adult body condition were not supported in the most parsimonious models.

**Table 1:** Linear mixed-effects models of Cape gannet behavioural parameters.

Model	Chick age	Sex	Initial time	BC	Sex∶Chick age	Sex∶BC	np	AIC	ΔAIC
Foraging trip duration									
T1	•	•			•		7	13553.3	0.0
T2	•	•		•	•		8	13557.8	4.5
T3	•	•		•	•	•	9	13560.1	6.8
T4	•	•					6	13560.7	7.4
T5	•	•		•			7	13564.8	11.5
T6	•	•		•		•	8	13566.7	13.4
Nest attendance duration									
N1	•		•				28	11798.0	0.0
N2	•		•	•			29	11800.1	2.1
N3	•	•	•				29	11801.1	3.1
N4	•	•	•	•			30	11803.2	5.2
N5	•	•	•	•		•	31	11805.5	7.5
N6	•	•	•		•		30	11808.1	10.1
Provisioning rate									
Tf1	•	•	-				6	5752.8	0.0
Tf2	•		-				5	5753.1	0.3
Tf3	•	•	-	•			7	5760.1	7.2
Tf4	•		-	•			6	5760.2	7.4
Tf5	•	•	-	•		•	8	5765.8	12.9
Tf6	•	•	-		•		7	5766.7	13.9

These were constructed using log-transformed foraging trip duration (T), nest attendance duration (N) and provisioning rate (Tf) of breeding adult birds at Bird Island, Algoa Bay, as a function of predictor variables: chick age as a continuous number, adult sex, adult sex interacting with chick age, time of bout initiation, adult body condition index (BC) and BC interacting with sex. Number of parameters in each model (np), Akaike Information Criterion (AIC) scores and the AIC difference from the most parsimonious model (ΔAIC) are shown.

**Figure 1:**
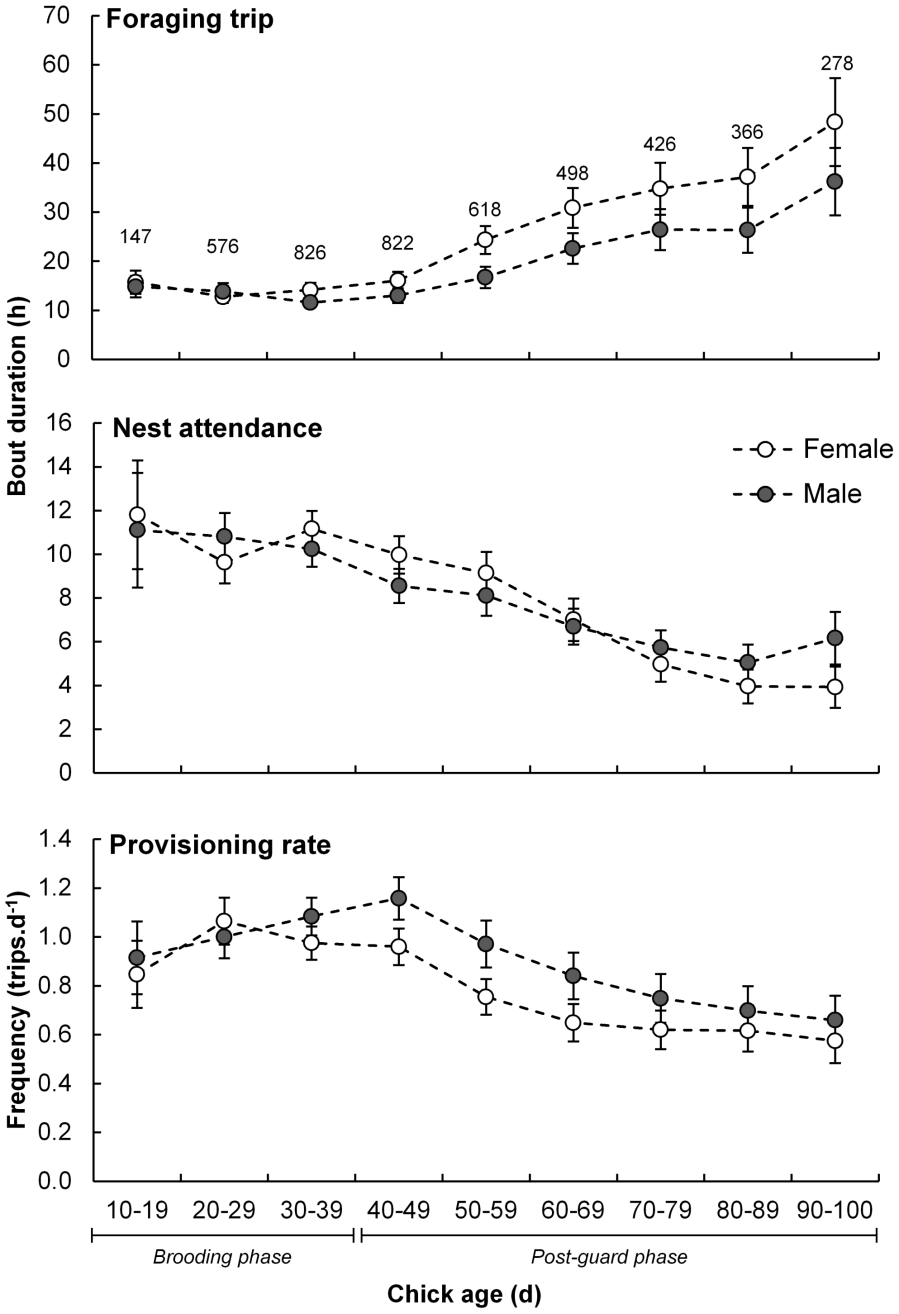
Sex-specific behavioural parameters of adult Cape gannets at Bird Island, Algoa Bay, as a function of the age of the chicks they are provisioning. Females are represented by white circles and males by dark circles. Total number of foraging trips recorded per chick age category, as well as brooding and post-guard phase of chicks, are indicated.

Foraging trip duration increased with chick age (Fig. 1; Table 2). This was more pronounced after the chick was older than 50 days (Fig. 1), where prior to this the linear trend respective to chick age was not significant (F_(1,1957)_ = 0.49, p = 0.48). Nest attendance duration decreased with chick age (Fig. 1; Table 2). Provisioning rates were highest when chicks were of an intermediate age and least frequent near fledging, showing an overall decreasing linear trend with chick age (Table 2).

**Table 2:** Most-parsimonious linear mixed-effects models of Cape gannet behavioural parameters fitted by restricted maximum likelihood.

		Foraging trip duration (h)	Nest attendance (h)	Provisioning rate (trips.d^−1^)
	df	P (SE)	P (SE)	P (SE)
Intercept	*1*	1.78 (0.07)	1.45 (0.54)	0.74 (0.02)
Chick age	*1*	0.02 (0.001)	−0.02 (0.001)	−0.004 (0.0003)
Sex^M^	*1*	0.11 (0.09)	-	0.06 (0.02)
Time	*23*	-	see Figure 4	-
Body condition index	*1*	-	0.16 (0.09)	-
Sex^M^∶Chick age	*1*	−0.01 (0.002)	-	-

M: Coefficient reflective of male behaviour.

These used the logarithm of foraging trip duration, nest attendance duration and provisioning rate of breeding adult birds at Bird Island, Algoa Bay, as a function of chick age as a continuous number, adult sex, adult sex interacting with chick age, time of bout initiation and adult body condition index (Model T1, N2 and Tf1; Table 1). Model parameter estimates (P) represent the directional effect of the predictors relative to their reference category. Time is a factor divided into one-hour bins.

**Figure 2:**
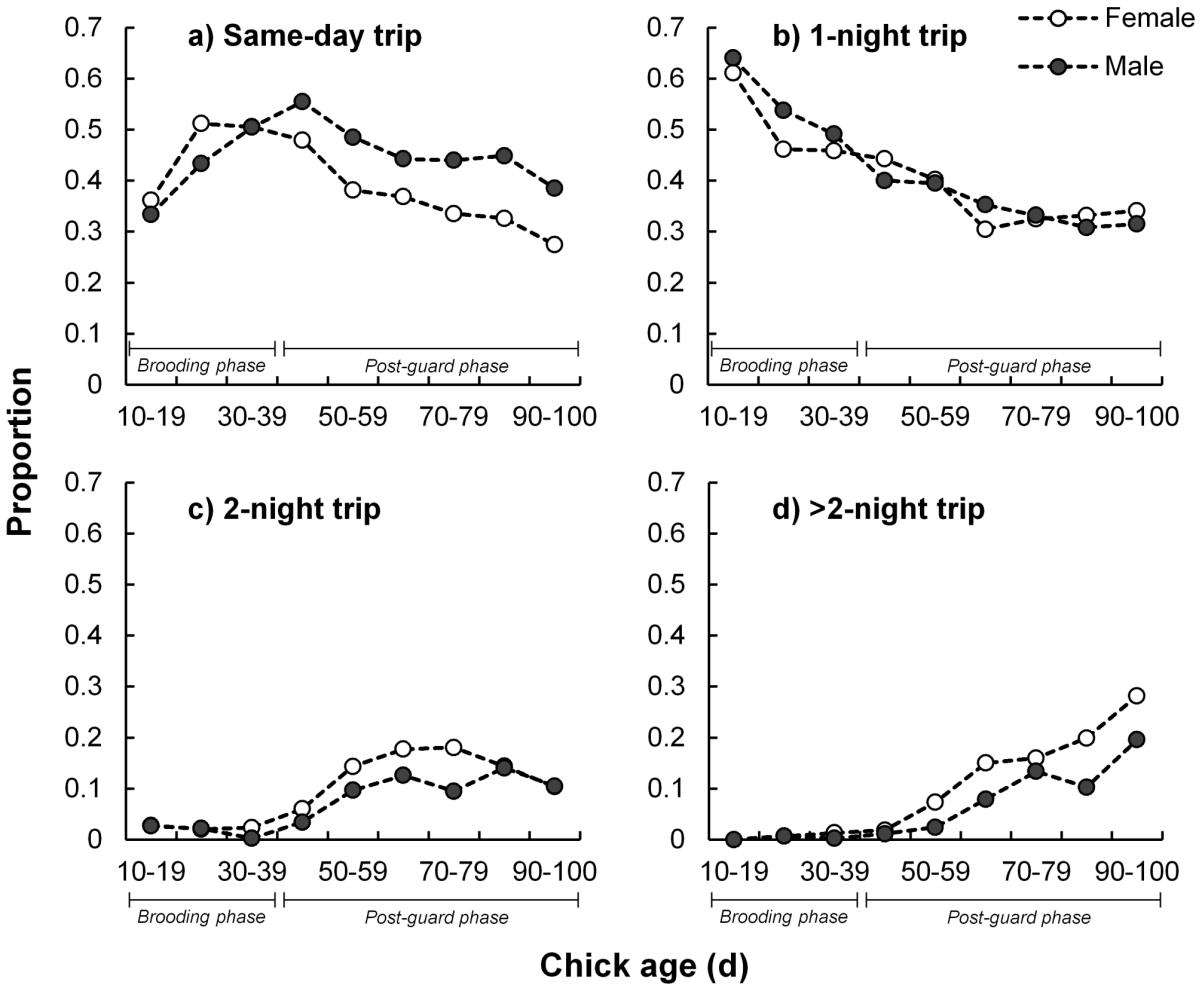
Sex-specific foraging trip length proportions of adult Cape gannets at Bird Island, Algoa Bay as a function of chick age. Same-day (a), one- (b), two- (c) and greater than two-night (d) foraging trips are indicated for female (white circles) and male (dark circles) birds. Brooding and post-guard phase of chicks are indicated.

Foraging trip duration was related to adult sex interacting with chick age with females spending longer periods at sea than males (average over chick-rearing period of 24.3±3.4 versus 17.7±1.4 h, respectively; Fig. 1), especially when the chicks neared fledging (Table 2). Males made more frequent foraging trips than females (0.95±0.07 versus 0.84±0.07 trips.d^−1^; Fig. 1; Table 2). Furthermore, males made a greater proportion of same-day foraging trips than females and this was especially apparent amongst adults attending advanced chicks (Fig. 2; Table 2). Nest attendance duration did not appear to be a function of sex (Table 1) although males generally spent longer periods at the nest (9.5±1.1 versus 8.7±1.1 h; Fig. 1).

**Figure 3:**
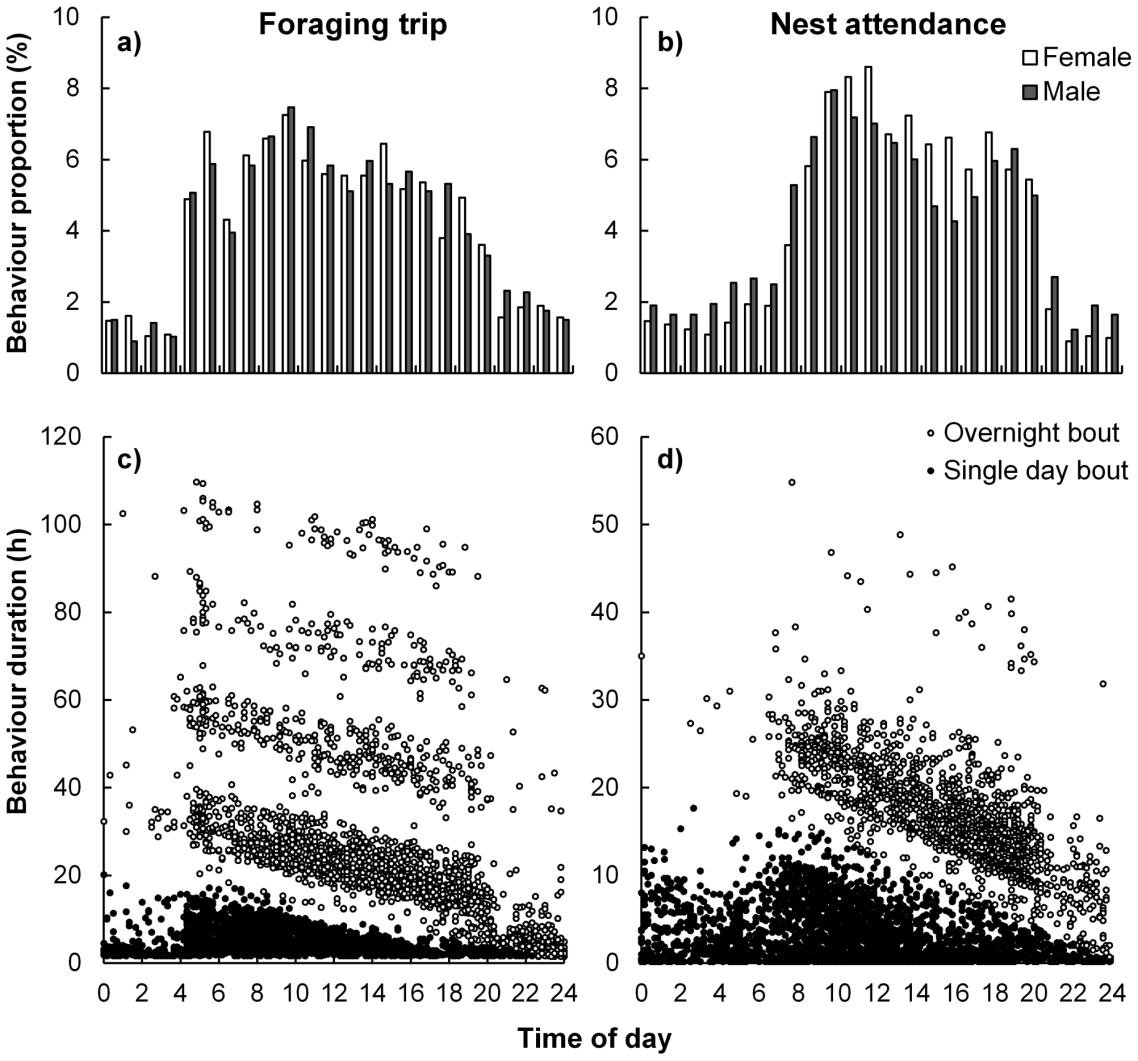
Timing of bout initiation and duration for breeding Cape gannets at Bird Island, Algoa Bay. Proportion (a and b) and duration (c and d) of foraging trip (left) and nest attendance (right) bouts as a function of bout initiation time are indicated. Behaviour proportions are distinguished by sex (males as dark bars and females as white) and behaviour durations according to those which occurred over one or more midnights (white circles) and those which did not extend over any midnights (dark circles). Trip duration is shown for trips which occurred over fewer than five nights (98.4% of all trips).

Sex-specific interactions with nest departure and arrival time were not useful in explaining variability in foraging trip or nest attendance durations. Nest departure time peaked between 09:00 and 10:00 (Fig. 3a). Females tended to arrive back at their nests later (peaking between 11:00 and 12:00) than males (peaking between 09:00 and 10:00; Fig. 3b).

**Figure 4:**
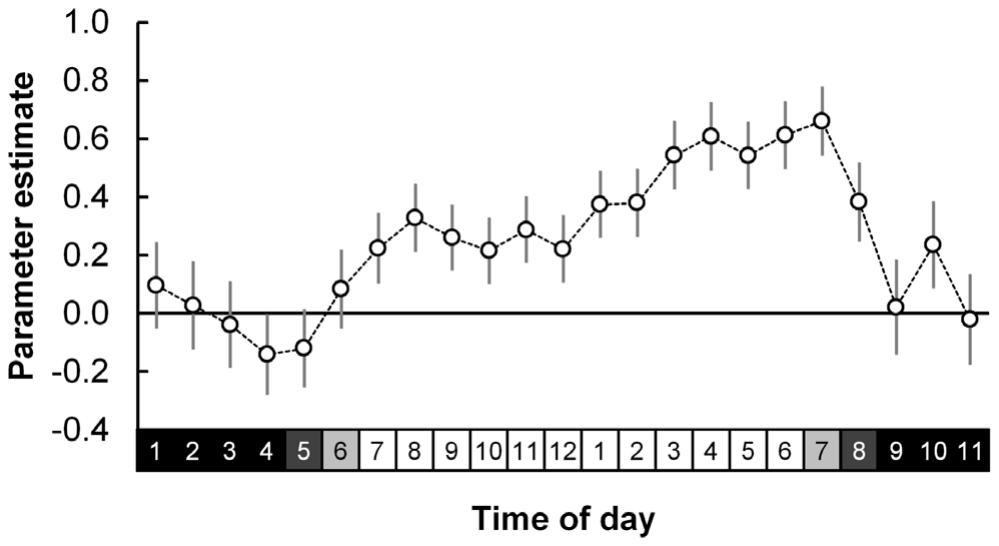
Parameter estimates of nest arrival time as a predictor of nest attendance duration for breeding Cape gannets at Bird Island, Algoa Bay. Time is modelled as 24 one-hour bins. Parameter estimates are derived from Model N2, Tables 1 and 2. Standard error bars as well as day (light squares) and night (dark squares) are indicated.

As expected, nest arrival time was a highly significant predictor of overall adult nest attendance duration (Table 2; Fig. 4). Birds departing earlier or arriving later generally had longer foraging trip or nest attendance bouts, respectively, irrespective of the number of nights away from or at the nest (Fig. 3c and d; Fig. 4). Departure frequency noticeably increased (between 04:00 and 05:00) after civil dawn (when the sun is below 6° on the horizon), tapering off by 06:00 (roughly 1.5 h after sunrise) and then increasing to a relatively steady level by 07:00 when arrivals to the nest started increasing (Fig. 3a and b). Trips which were initiated between dusk and dawn (n = 561; 12.3%) were shorter (8.5±1.5 h) than those initiated during daylight hours (22.3±0.8 h) and more frequent following sunset than before sunrise (Fig. 3a).

There was no significant difference in body condition between males and females at the time of VHF deployment (5.89±0.13 versus 5.90±0.12 g.mm^−1^; t_(2,98)_ = 0.04, p = 0.92), and consequently sexual segregation in adult body condition was not a predictor of adult behavioural parameters (Table 1). Additionally, both overall foraging trip duration and provisioning rate did not appear to be driven by adult body condition (Table 1). However, gannets which were in better body condition spent longer periods of time at their nests (Table 2).

## Discussion

Using an extensive data set on foraging trip and nest attendance durations it was here clearly demonstrated that time-activity budgets in a chick-rearing seabird were sex-dependent and linked to chick development. Furthermore, the amount of time that adults were spending at their nests was related to the time of nest arrival, while nest attendance was positively related to body condition. With one exception [27], this represents the first comprehensive analysis of continuous time-activity budget data for a volant seabird through chick development up to fledging. Unlike the study on wandering albatrosses, *Diomedea exulans* [27], we used coded (rather than multiple-frequency), leg-ring-attached (rather than temporary, tail-feather attachment) VHF transmitters, allowing for fine-scale simultaneous sampling of a large number of study birds which yielded high-resolution, long-term nest attendance data [24].

### Sex-related differences

In monomorphic species, inter-sex differences in foraging behaviour are thought to be related to intraspecific competition and resource partitioning or parental investment strategies and requirements [9], [55]. Sexual segregation in foraging effort is apparent for both the western [5], [25] and southern coast (this study) Cape gannet populations. Longer foraging trip durations by female Cape gannets have been demonstrated before from South Africa's west coast populations [5], [25]. However, foraging trip duration decreased with increasing chick age [5] whereas an opposite trend was evident from the current study (Fig. 1). As the current study's data extend until fledging, an advantage of VHF-based monitoring [24], the younger chick age at which monitoring ceased [5] likely yielded this difference. Foraging trip duration did not increase significantly prior to chicks reaching 50 days of age (Fig. 1), which was the approximate age at which the west coast study stopped monitoring [5]. Direct monitoring of seabird nests often ceases during the post-guarding phase when adults only return briefly to provision their frequently non-attended chicks (e.g. [5], [8]). During this period the likelihood of not observing birds during provisioning bouts increases. These results therefore present a new insight into seabird foraging ecology in the poorly-documented post-guarding stage of a chick's development.

Data from this study supports the existence of sexual segregation in parental investment strategies in seabirds, particularly as the chick ages. Although females generally return to their nests later than males (Fig. 3b), perhaps suggesting differing foraging locations, it is unlikely that intraspecific competition entirely drives this segregation as females do not have consistently longer foraging trip durations than males (Fig. 1) (*sensu* [9]). Northern gannets, *Morus bassanus* do not show sexual dimorphism in foraging trip duration, but are similar to Cape gannets in that both species show sex-specific time-budgets at sea [8], [25]. Female northern gannets forage in different areas, make longer, deeper dives and spend more time resting on the sea surface than males [8]. This suggests that female gannets may utilise different resources compared to males to reduce intraspecific competition [5], although not demonstrated in this study.

The cost of bearing an egg may be associated with the longer time that females spend away from the nest and their need to replenish spent resources [15], [25], [56]. In some alcids the male is solely responsible for chick provisioning in the latter stages of chick development [11], [55] whereas in rockhopper penguins, *Eudyptes chrysocome* the male effectively fasts during brooding while the female provisions their chick and possibly herself [57]. Female Cape gannets have a greater proportion of longer foraging trips, especially when the chick is older (Fig. 2; Table 2), suggesting that they might use this period and strategy to replenish their spent resources associated with egg development [15], [58]. In light of this, the disparity in foraging duration would possibly be accentuated through a reduction in prey availability following high levels of prey consumption during the advanced stage of the breeding season [48], [59]. Additionally, male Cape gannets appear to work harder towards their chick's development by foraging more frequently than females as their chick ages (Figs. 1 and 2; although this does not indicate that the quantity of food provisioned differed between parents as no data was collected on meal size). This has been demonstrated in other bird taxa where the costs of egg production are shifted towards male-dominated chick provisioning [11], [13]. Males also spend more time at the nest with the chick while their partner forages, especially when they are in good body condition.

### Other drivers of time-activity budgets

The energetic requirements of developing chicks generally impact on foraging behaviour in seabirds [2], [3], [34], [60]. Adult seabirds must increase their foraging effort and provisioning to meet the demands of their chicks for increasing amounts of food [60], [61]. Local prey may potentially be depleted as the breeding season progresses and force seabirds to increase their foraging effort in search of more distant prey [39], [48], [59], [60]. Additionally, following the brooding phase, when parents often leave their chicks unattended, the number of conspecific foragers at sea increases substantially, increasing the density of birds in the local marine environment. This might enable birds to maintain an efficient foraging network over larger distances [40], [62]–[64]. It is therefore likely that the observed increase in foraging effort (longer trip durations) with chick age is as a response to either or both increased chick demand and local prey depletion or accessibility during the breeding season. Regular acoustic-based prey abundance assessments would be able to assess whether local prey availability diminishes significantly at this site during the course of the Cape gannet breeding season.

Foraging behaviour amongst seabirds is intrinsically linked to body condition [14], [65]. Therefore Cape gannets, which were in better condition, expectedly spent more time at their nests, reflecting more discretionary time in their time budget [36], [66]. However, there appeared to be no effect of body condition on foraging trip duration or frequency. This assumed that the single measurement of body condition at the time of transmitter deployment was representative of that adult's relative condition during the entirety of chick provisioning. However, body condition changes throughout the breeding season [5] and therefore these data may not be sensitive to intra-seasonal fluctuations in body condition. Another method of body condition calculation, for example fat deposit measurement using ultra-sound (e.g. [67]), or a continuous measure of adult body condition throughout the breeding season such as automated body mass measurements (e.g. [68]), while minimising handling and disturbance [69], might be more informative.

The time when Cape gannets leave or arrive at their nests constrains foraging trip and nest attendance duration. Birds leaving earlier in the day had more daylight hours available for same-day foraging and those arriving later tended to remain at their nests overnight (Fig. 4), therefore reflecting longer overall foraging trips and nest attendance bouts on average. Sulids have been shown to work harder by flying and diving more often while foraging when less daylight hours are available [43] thus shortening their overall foraging trip. Foraging trips are also extended to an overnight strategy after an unsuccessful first day's foraging or to reach more distant profitable foraging grounds [70], [71]. In this study, the number of nights away from the nest adds to the total foraging duration such that clearly multimodal-trip durations, as have previously been recorded for northern gannets [71], were apparent (Fig. 3c). Although not presented in this study, preliminary inter-seasonal data from the VHF system (GMR, unpubl.) showed a clear temporal trend in peak departure and arrival times to and from the island, likely correlated with seasonal changes in dawn and dusk times. This study did not incorporate this seasonal trend as the majority of foraging trips recorded occurred around the summer solstice (82% recorded within ±40 days of 20–23 December), when daylight hours varied little (39 minutes or less variation). Cape gannets do not forage at night [70], but it seems that they do occasionally depart their nests (12.3% of foraging trips) well after the sun has set. These observations need further verification through nocturnal direct observations of nest attendance, but it is not uncommon to observe a flying Cape gannet illuminated by the lighthouse beam on Bird Island several hours after sunset (pers. obs.). Some seabirds (e.g. Procellariiformes), historically viewed as being visual foragers [72], do occasionally return to or depart from their nests after dark [27] or use other cues (such as olfaction) to locate their prey [73], [74]. Additionally, while not tested in this study, moon phase can have a noticeable influence on nocturnal activity patterns in seabirds [75]. The visually-foraging Cape gannet might utilise nocturnal hours to commute between nearby profitable foraging grounds, thus maximising available daylight hours for active foraging.

### Conclusions

The chick-provisioning strategy observed in this study demonstrates an example of gender role shifts where the paternal partner increases its relative reproductive effort as a response to decreased investment by females as offspring near independence. In addition to sex, the developmental stage of the chick largely influenced foraging effort while adult body condition determined the amount of time allocated to nest attendance. The effect of resource availability on animal breeding behaviour [21] is important in seabirds because their behaviour can be used to indicate the state of prey [31], [76]. This has relevance to fisheries management as behavioural attributes of seabirds can be used as a tool for monitoring prey availability during chick-rearing [2], [76]. Clearly a good understanding of the full spectrum of intrinsic and extrinsic influences of seabird behaviour would need to underpin such an approach. The automated VHF system provides a continuous measure of colonial animal behaviour on which the effects of predictor variables can be tested [24]. Linking fine-scale time-activity budget data to acoustic survey data on epipelagic prey, while accounting for the influences of sex and chick developmental stage, would be the next step forward.
